# Efficacy of virtual reality therapy in ideomotor apraxia rehabilitation: A case report

**DOI:** 10.1097/MD.0000000000026657

**Published:** 2021-07-16

**Authors:** Wookyung Park, Jongwook Kim, MinYoung Kim

**Affiliations:** a Department of Rehabilitation Medicine, CHA Bundang Medical Center, CHA University School of Medicine, Gyeonggi-do, Korea; b Rehabilitation and Regeneration Research Center, CHA University School of Medicine, Gyeonggi-do, Korea.

**Keywords:** ideomotor apraxia, occupational therapy, rehabilitation, stroke, virtual reality

## Abstract

**Rationale:**

We report the possible therapeutic efficacy of immersive virtual reality (VR) rehabilitation for the treatment of ideomotor apraxia in a patient with stroke.

**Patient concerns:**

A 56-year-old man with sudden weakness of his left side caused by right frontal, parietal, and corpus callosal infarction was transferred to rehabilitation medicine center for intensive rehabilitation. Although his left-sided weakness had almost subsided 10 days after the onset of symptoms, he presented difficulty using his left hand and required assistance in most activities of daily living.

**Diagnoses:**

Ideomotor apraxia in a patient with right hemispheric infarction.

**Interventions:**

VR content was displayed to the study participants using a head-mounted display that involved catching of moving fish in the sea by grasping. Before and after of rehabilitative intervention including VR, functional measurements incorporating the Test of Upper Limb Apraxia (TULIA) were conducted. To directly compare therapeutic potencies under different conditions, success rates of consecutive grasping gesture performance were observed in VR, conventional occupational therapy setting, and augmented reality intervention.

**Outcomes:**

The patient demonstrated remarkable amelioration of apraxic symptoms while performing the task in the VR environment. At 1 and 3 months after the training, he showed significant improvement in most functions, and the TULIA score increased to 176 from 121 at the initiation of therapy. The number of successful grasps during 30 trials of each grasp trial was 28 in VR, 8 in the occupational therapy setting, and 20 in augmented reality.

**Lessons:**

This case report suggests the possible therapeutic efficacy of immersive VR training as a rehabilitative measure for ideomotor apraxia.

## 1 Introduction

Ideomotor apraxia refers to the inability to mimic limb gestures, despite the absence of paralysis.^[1]^ It is a common therapeutic target in patients undergoing brain damage rehabilitation.^[2]^ Although patients understand the actions to be performed, they cannot translate it to actual movement.^[3]^ The primary disability in patients with apraxia is an “automatic-voluntary dissociation,” which is the inability to perform voluntary movements, although the patient may carry out such movements.^[4]^ These patients are unable to initiate automatic motor responses in real-world rehabilitation settings. Virtual reality (VR) is a form of information and communication technology that displays simulated environments and interactions with the user. It has shown promising results in rehabilitation therapy by improving patients’ motivation and willingness to participate in.^[5]^ Among the domains of VR training for patients with stroke, functional recovery of the upper extremity was reported to be most significant.^[6]^ Rehabilitation for the treatment of apraxia should include facilitation of automatic responses, which may be induced by VR. We present a case of dramatic amelioration of apraxic symptoms in a patient with stroke when immersive VR was applied. The immediate responses of automatic-voluntary dissociation were compared in three different settings, and final functional improvements after VR training rehabilitation and changes in the Test of Upper Limb Apraxia (TULIA) score are presented.

## 2 Case presentation

### 2.1 Patient's characteristics

A 51-year-old right-handed man presented with sudden weakness of his left side and was diagnosed with right frontal, parietal, and corpus callosal infarction. He was transferred to the Department of Rehabilitation Medicine 10 days after the onset of symptoms, and his left-sided weakness had subsided (≥ fair plus, using the Medical Research Council Muscle scale).^[7]^ As for gross motor ability, he could stand independently but could not walk. Despite the absence of remarkable paralysis, he experienced difficulty using his left hand and required assistance in most activities of daily living (ADL). The scores of the Mini-Mental State Examination, Montreal Cognitive Assessment, and intelligence quotient of Wechsler Adult Intelligence Scale-IV were 26, 23, and 80, respectively, showing fair cognitive function. He could not mimic some gestures after verbal instructions; however, he could understand and explain the motor sequences required for those movements. For example, he could not grasp the examiner's hand when verbally instructed and required a long time to shake the examiner's hand. After initiation of the grasping hand movement, his hand muscles showed excessive rigidity, and he was unable to control the movement and release the hand. The TULIA score confirmed apraxia (score 124 on 240 points).^[8]^ During the TULIA assessment, the score of the imitation part was higher than that of pantomime. For example, the item “put the index finger on top of the nose” was scored as 4 out of 5, while the scores for items “comb hair” and “drink from a glass” were lower with 2 out of 5. Within imitation and pantomime, a descending trend was observed with the highest score in non-symbolic and intransitive, and lowest in transitive movements. Errors in movement were observed in every task of TULIA, and the intended gestures were not achieved. Written informed consent was obtained from the patient. This study was approved by the institutional review board of the study hospital.

### 2.2 Acquisition of brain magnetic resonance imaging (MRI) and diffusion tensor images analysis

**Figure 1:**
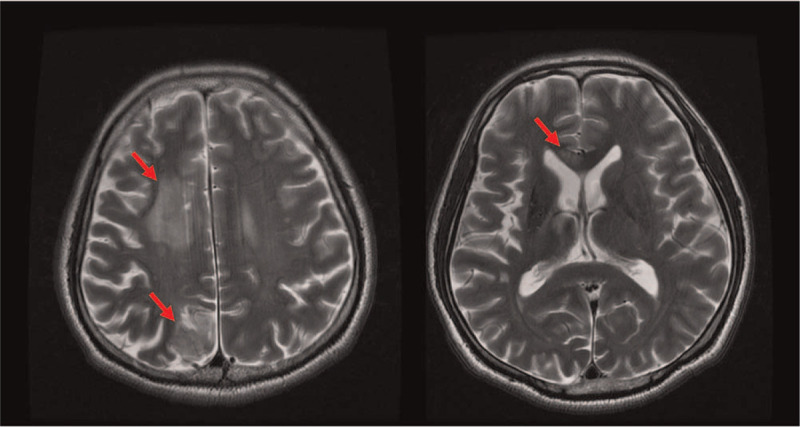
T2-weighted axial brain magnetic resonance images show subacute to chronic infarction (red arrow) in the right frontal lobe (superior frontal and precentral gyri), right parietal lobe, and right corpus callosum.

Brain MR images were acquired using a 3T GE Signa System (General Electric, Milwaukee, WI) at 1 month after onset. Axial T2-weighted, axial T1-weighted fast spoiled gradient recalled echo and diffusion tensor images (DTI) sequences were included. T1, T2-weighted showed subacute to chronic infarction in the right corpus callosum, right frontal lobe (superior frontal and precentral gyrus), and right parietal lobe. It also showed old infarction in the right centrum semiovale and basal ganglia with ipsilateral Wallerian degeneration (Fig. 1). DTI acquisition parameters were as follows: repetition time = 13,000 ms, echo time = 108.6 ms, field of view = 51.2 × 51.2 cm^2^, matrix size = 256 × 256, slice thickness = 2.0 mm. DTI data were analyzed using DSI studio software (http://dsi-studio.labsolver.org). Regions of interests were defined automatically based on an anatomical atlas loaded into the DSI studio program. The fractional anisotropy (FA) values, tract volume, and number of fibers were obtained. Analysis results revealed decreased value of FA in the right cingulate gyrus, superior longitudinal fasciculus (SLF) I, and decreased value of tract volume and number of fibers in the right SLF II, SLF III, uncinate fasciculus, and corticospinal tract compared with the left side^[9]^ (Table 1). The FA decrement results could be interpreted as the cerebral infarction impact on these fibers.

**Table 1:** Diffusion tensor tractography parameters.

	FA		Tract volume, mm^3^		Number of fibers	
Tract	Right	Left	Right	Left	Right	Left
Cingulate gyrus	0.351	0.512	2499.6	2454.5	606	504
ILF	0.601	0.513	1019.9	653.1	62	50
SLF I	0.266	0.405	211.9	24.1	28	1
SLF II	0.415	0.426	276.3	5704.8	20	1118
SLF III	0.437	0.471	3700.1	4186.1	678	1416
Uncinate fasciculus	0.519	0.475	2703.9	5744.4	380	935
Corticospinal tract	0.447	0.510	26.4	722.1	1	27
Corticothalamic pathway	0.498	0.477	1782.0	2654.1	171	282

FA = fraction anisotropy, ILF = inferior longitudinal fasciculus, SLF = superior longitudinal fasciculus.

### 2.3 Rehabilitative intervention including a virtual reality training

**Figure 2:**
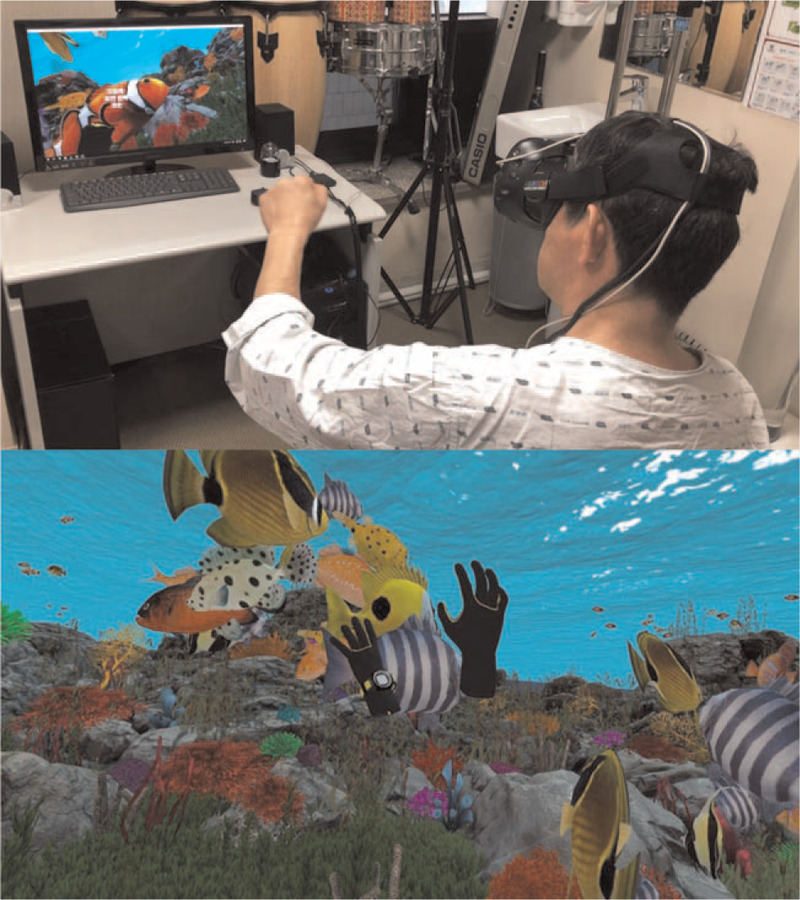
A representative scene of rehabilitative training using virtual reality with a head-mounted display while the patient is using his left hand (top). A screenshot of the software (bottom).

The patient was trained with repeated practice of ADL tasks (eating, grooming, and dressing), which involved grasping and releasing actions of the hands. Passive cueing was provided with physical guidance or verbal instructions for motor sequences. However, the progress of learning was delayed for sequential activities in most ADL. He received VR training as well as conventional occupational therapy (OT), which included training in reaching, grasping, manipulation, and releasing objects and practices of dressing, eating, drinking, washing, brushing teeth, combing hair, etc. The VR training system adopted a head-mounted display, Vive (HTC Inc., Taiwan), which uses the “Ocean Catch” software (Hancom Malang Malang VR, Korea), ideated by the corresponding author of this report. This immersive VR content simulated an under the sea virtual environment and provided opportunities for grasping movements to catch moving fish (Fig. 2). The corresponding occupational therapist assisted the patient in the VR training. Each VR training session was initiated with visual and narrative auditory cues that directed the number of fish to be caught. The VR system had three levels of difficulty with regard to size, swimming speed, and the number of fish to be caught. The levels were adjusted appropriately. Usually, each task required 3 to 5 min, and feedback by displaying a success score was provided upon task completion. The patient received VR training for 20 min/day, 5 days/week for 4 weeks. He stated that VR training enhanced his upper extremity function, and the therapy itself was interesting. Although adverse events were monitored at each VR session, he developed motion sickness during the first session, which resolved within a few minutes. Subsequently, the VR training was continued without interruption in the session and the previously mentioned adverse event did not recur until the last training session.

### 2.4 Immediate automatic responses and improvements in apraxic symptoms

After 4 weeks of VR training, the TULIA score increased to 161 from 121 at the initiation of the therapy in both the pantomime and imitation domains (Table 2), and errors in performing simple grasp-and-release tasks did not completely disappear. Four weeks after the initiation of rehabilitation, he scored 84 in the Korean Version of the Modified Barthel Index (MBI), which was an increase from an initial score of 55. Improvements were also observed in personal hygiene, bathing, toileting, dressing, stair climbing, ambulation, and transfer fields. At the follow-up visit, 16 weeks after training initiation, most of his apraxic symptoms were found to have resolved.

**Table 2:** Evaluation of patient function at baseline, 4 weeks, and 12 weeks following rehabilitation using VR.

Functional assessments		Baseline^∗^	After 4 wks	After 12 wks
Mini-mental status examination (30)		26	30	30
Hand function test					
Grip strength, kg	Rt/Lt	28/18	30/24	26/28
Lateral pinch, kg	Rt/Lt	7/6	8/7	6.5/6.5
Tripod pinch, kg	Rt/Lt	5/4	6/5	6/5
Manual function test (32)	Rt/Lt	30/20	31/28	31/28
Fugl-Myer assessment (66)	Rt/Lt	65/39	65/58	66/62
Modified Barthel index (100)		55	84	87
Test of Upper Limb Apraxia score					
Imitation, non-symbolic (40)		27	35	35
Imitation, intransitive (40)		22	27	33
Imitation, transitive (40)		19	23	28
Imitation subtotal (120)		68	85	96
Pantomime, non-symbolic (40)		22	31	31
Pantomime, intransitive (40)		17	23	27
Pantomime, transitive (40)		14	21	22
Pantomime subtotal (120)		53	75	80
Total (240)		121	160	176

The units and figures in parentheses refer to the unit for the strength of grasp and the full score for each evaluation, respectively.

∗ Ten days after onset of stroke, after 4 wks; immediately after 4 wks of training, after 12 wks; at follow-up via outpatient department.

### 2.5 Differences in performance by different conditions

Despite significant improvement in apraxia after 4 weeks of rehabilitation, he still showed residual symptoms. We wanted to see if immersive VR had an immediate effect on motor performance. For the assessment of apraxic symptoms, the number of successful grasps was counted from 30 trials of simple “consecutive grasp-and-release gestures,” which he had difficulty performing. “Grasp-and-release” was defined as full flexion of all fingers followed by their complete extension. For the next grasp, there had to be about one second of rest. The activity was performed following verbal instruction in three different settings: a real-world setting in OT receiving room, commercial non-immersive augmented reality (AR), and VR environments. For the real-world setting, the block manipulation activity was assessed. RAPAELSmart Glove (Neofect Inc., Korea) was used as the AR instrument. We observed an incomparably best motor response of the left hand during the VR condition. The number of successful maneuvers using OT, AR, and VR was 8 (26.7%), 20 (66.7%), and 28 (93.3%), respectively.

## 3 Discussion

The immersive VR training seemed to have significantly ameliorated ideomotor apraxia in the present case. Compared with other conditions, such as block manipulation in the real world and conducting an AR task using a smart glove, the patient showed immediate improvement in grasp and release performance in the VR condition. This means that the automatic motor response is more easily induced by the VR environment. Given the basic characteristics of apraxia (automatic-voluntary dissociation), its impact on daily life might be overlooked.^[10]^ However, the severity of apraxic symptoms correlates with reduced independence in daily living skills and reduced improvement in independent functioning.^[11,12]^ This case also showed improvement in ADL with an elevated MBI score along with a decrement in apraxic features that occurred during the VR training.

Apraxic symptom has been associated with damage to the parietal and frontal cortex as well as the white matter connections between them. Furthermore, anterior callosal lesions and basal ganglia were reported as the apraxia-related area.^[13]^ In the present case, we observed parietal and frontal cortex defects in the right hemisphere and corpus callosum. The reduced FA values and tract volume revealed partial injury in the right cingulate gyrus, SLF, and uncinate fasciculus, which contain projections between frontal and parietal cortex.^[9]^ These brain lesions seemed to cause apraxic symptoms in patients and might have affected the VR rehabilitation outcome in this case. The follow-up study of DTI, which identify for changes in values of FA, would be helpful to confirm the effectiveness of the VR rehabilitation.

VR enables repetitive practice, provides feedback on the performance, and motivates the user to endure the training for motor rehabilitation. Feedback on the performances given by the virtual environment would be stronger than that given during real-world practice.^[14]^ The VR used in this study also provided feedback immediately after each training session. Above all, it seems that the fun and motivation induced by the immersive VR may have strengthened the intensity of response to rehabilitation.^[15]^ According to a recent Cochrane review, VR may be beneficial for improving upper limb function and ADLs in post-stroke patients when used as an adjunct to usual care.^[6,16]^ This positive effect could have been induced by increased motivation of the patients while using VR. Therefore, we concluded that immersive VR provided a motivating condition that enabled the patient to overcome automatic-voluntary dissociation, which might be corrected only by activating the function at an unconscious level.

Consequently, his dexterity and ADLs improved over a relatively short period (4 weeks). This improvement persisted even 12 weeks after rehabilitation. Although the patient received additional conventional rehabilitation, considering the immediate responses to VR training, it is reasonable to conclude that VR training was beneficial in this case.

## 4 Conclusion

Currently, there is no specific treatment for patients with motor apraxia after stroke.^[17,18]^ A recent review suggested that rehabilitative training alone would be insufficient for a sustained benefit.^[19]^ However, our patient showed significant recovery from apraxia 3 months after completion of our intervention. More studies and large-scale clinical trials are needed to determine the therapeutic efficacy and its mechanism in VR application for apraxia. To the best of our knowledge, no studies have reported on the treatment of limb apraxia using immersive VR. This case report shows the beneficial effects of immersive VR-based rehabilitation in a patient with severe apraxia.

## Author contributions

**Conceptualization:** MinYoung Kim.

**Data curation:** Wookyung Park, Jongwook Kim.

**Supervision:** MinYoung Kim.

**Writing – original draft:** Wookyung Park.

**Writing – review & editing:** MinYoung Kim.
